# Impregnation of bone chips with alendronate and cefazolin, combined with demineralized bone matrix: a bone chamber study in goats

**DOI:** 10.1186/1471-2474-13-44

**Published:** 2012-03-24

**Authors:** Nina MC Mathijssen, Gerjon Hannink, Peter Pilot, B Wim Schreurs, Rolf M Bloem, Pieter Buma

**Affiliations:** 1 Department of Orthopaedics, Reinier de Graaf Groep, Reinier de Graafweg 3/11, 2625, AD Delft, The Netherlands; 2 Bislife, Galileiweg 8, 2333, BD Leiden, The Netherlands; 3 Orthopaedic Research Laboratory, UMC St. Radboud, P.O. Box 9101, 6500, HB Nijmegen, The Netherlands; 4 Department of Orthopaedics, UMC St. Radboud, P.O. Box 9101, 6500, HB Nijmegen, The Netherlands; 5 Department of Operating Rooms, UMC St. Radboud, P.O. Box 9101, 6500, HB Nijmegen, The Netherlands

## Abstract

**Background:**

Bone grafts from bone banks might be mixed with bisphosphonates to inhibit the osteoclastic response. This inhibition prevents the osteoclasts to resorb the allograft bone before new bone has been formed by the osteoblasts, which might prevent instability. Since bisphosphonates may not only inhibit osteoclasts, but also osteoblasts and thus bone formation, we studied different bisphosphonate concentrations combined with allograft bone. We investigated whether locally applied alendronate has an optimum dose with respect to bone resorption and formation. Further, we questioned whether the addition of demineralized bone matrix (DBM), would stimulate bone formation. Finally, we studied the effect of high levels of antibiotics on bone allograft healing, since mixing allograft bone with antibiotics might reduce the infection risk.

**Methods:**

25 goats received eight bone conduction chambers in the cortical bone of the proximal medial tibia. Five concentrations of alendronate (0, 0.5 mg/mL, 1 mg/mL, 2 mg/mL, and 10 mg/mL) were tested in combination with allograft bone and supplemented with cefazolin (200 μg/mL). Allograft not supplemented with alendronate and cefazolin served as control. In addition, allograft mixed with demineralized bone matrix, with and without alendronate, was tested. After 12 weeks, graft bone area and new bone area were determined with manual point counting.

**Results:**

Graft resorption decreased significantly (p < 0.001) with increasing alendronate concentration. The area of new bone in the 1 mg/mL alendronate group was significantly (p = 0.002) higher when compared to the 10 mg/mL group. No differences could be observed between the group without alendronate, but with demineralized bone, and the control groups.

**Conclusions:**

A dose-response relationship for local application of alendronate has been shown in this study. Most new bone was present at 1 mg/mL alendronate. Local application of cefazolin had no effect on bone remodelling.

## Background

Different types of bone grafts from bone banks are widely used for the restoration of bone defects in clinical practice with satisfying clinical outcomes [1,2]. In addition, allograft bone bank chips are used during bone impaction grafting, a well-accepted and clinical successful procedure for the restoration of bone stock loss around loose implants [3-5]. This procedure is hence applied during revision arthroplasty. A possible problem associated with the use of bone grafts in loaded conditions, like in revisions, is a too fast resorption of graft material. Accelerated bone graft resorption before the formation of new bone may cause loss of stability, ultimately resulting in failure of the revised hip or knee implant [6-8].

Bisphosphonates might reduce bone graft resorption by inhibition of the osteoclasts [9]. Bisphosphonates have a high affinity for Ca^2+^, and therefore are targeted to areas of high bone turnover and adhere closely and selectively to sites of active bone remodelling. Osteoclasts trying to resorb the bone, release the bisphosphonates from the bone mineral. Bisphosphonates become internalized in the osteoclast, resulting in apoptosis of the osteoclast [10].

Although bisphosphonates efficiently suppress bone resorption by apotosis of osteoclasts, systemic treatment will only reach the revascularized parts of the bone graft and the concentration of bisphosphonates in regions of the skeleton with low blood perfusion, for example the femoral neck, will be very low [11-13]. Therefore, in several studies a bisphosphonate solution was administered locally, with varying results [9,12,14-17]. In these studies, it was hypothesized that local application of bisphosphonates would decrease resorption after implantation of a new prosthesis. This might prevent mechanical instability and therefore provide better support during the early process of healing after the operation. This way, bone resorption and bone formation might be more balanced. Baas et al. [16] were not able to change this balance between bone formation and bone resorption. The bisphosphonate virtually blocked bone metabolism in their study. In addition, Jakobsen et al. [15] found a substantially decreased biomechanical implant fixation when the impacted morselized allograft had been soaked in bisphosphonate. On the contrary, it has been shown that local bisphosphonate treatment can protect the graft from resorption during the early postoperative period [9,12]. It has been suggested that bisphosphonates not only inhibit osteoclastic activity, but also might have a decreased anabolic or even a toxic effect on osteoblasts; a too high concentration of the unbound bisphosphonate may not only inhibit the osteoclasts but also the osteoblasts, and thus reduce new bone formation [18]. It is therefore important that the concentration of unbound free bisphosphonate is below the level in which osteoblasts are inhibited [9,15,16,19,20].

The chance of failure of a reconstruction is greatest immediately after surgery, when the graft bone has not been incorporated yet. This critical period after reconstruction with bone impaction grafting might be shortened when demineralized bone matrix (DBM) is used. DBM is a product of processed allograft bone and contains collagen, proteins and growth factors [21]. Demineralization of bone theoretically facilitates the release of growth factors which could increase the bioactivity of the graft and positively influence new bone formation and thus the amount of new bone [22,23] which might be of importance during the early critical period.

Another devastating complication following joint replacement surgery is infection. Systemic administered antibiotics are widely used to prevent and cure infection, however these antibiotics cannot easily reach the infected bone in an avascular area [24]. In addition, formation of a biofilm on the surface of the implant makes the systemic administered antibiotics less effective [25]. A solution to achieve high local antibiotic concentrations is to impregnate bone allograft chips with antibiotics. Several studies have shown that morselized bone can act as a carrier for antibiotics [26-32]. These studies show, both *in vitro *as *in vivo*, that bone grafts impregnated with antibiotics can be used as a prophylaxis against infections. However, the effectiveness of bone allograft healing in the presence of high levels of antibiotics has yet to be determined.

In this study we combined the local application of bisphosphonate, DBM and antibiotics with allograft bone chips in a bone chamber. The purpose of this study was to investigate the dose-response relation of alendronate-impregnated allograft with respect to the amount of graft bone and the amount of new bone or total bone after 12 weeks. We investigated whether locally applied alendronate has an optimum dose with respect to bone resorption and net bone formation. The second question of this study was whether the addition of DBM to bone chips impregnated with bisphosphonate would stimulate bone formation. Finally, we investigated whether the local application of antibiotics has an effect on bone remodelling.

## Methods

### Study design

25 mature Dutch female goats (Capra Hircus Sana, range 49-70 kg) bilaterally received four bone chambers in the cortical bone of the proximal medial tibia. The side and position of implantation of the 8 chambers were alternated with a random start. The bone conduction chamber consists of a titanium screw with a cylindrical interior space [33]. It is made up of two threaded half-cylinders held together by a hexagonal closed screw cap. The interior of the chamber has a diameter of 2 mm and a length of 7.5 mm. There are two openings at the end of the chamber to enable bone ingrowth. The threaded end of the implant is screwed into the tibia of the goat, so that the ingrowth openings are in direct contact with the endosteal transition from marrow into bone. Originally developed as a rat model, the bone conduction chamber was adjusted for use in goats [34]. Since the tibial cortex in rats is thinner than in goats, a 1-mm thick plate was inserted into the cap to lower the ingrowth openings through the cortex.

Five concentrations of bisphosphonates (0, 0.5, 1, 2, and 10 mg/mL alendronate, Fosamax, MSD) were combined with allograft bone supplemented with antibiotics (200 μg/mL cefazolin) (ALL + AB, ALL + AB + 0.5BIS, ALL + AB + 1BIS, ALL + AB + 2BIS, and ALL + AB + 10BIS). Allograft not supplemented with alendronate and cefazolin served as control (ALL). In addition, allograft mixed with DBM with alendronate (1 mg/mL) (ALL + DBM + BIS) and without alendronate (ALL + DBM) were tested (Table 1). The observation time was 12 weeks.

**Table 1:** Overview of experimental design

Group	Alendronate (mg/mL)	Cefazolin (μg/mL)	DBM
ALL	-	-	-

ALL + AB	-	200	-

ALL + AB + 0.5BIS	0.5	200	-

ALL + AB + BIS	1	200	-

ALL + AB + 2BIS	2	200	-

ALL + AB + 10BIS	10	200	-

ALL + DBM	-	-	+

ALL + DBM + BIS	1	-	+

Our sample size calculation was based on the results of a study of Aspenberg and Astrand [9]. The minimal relevant difference of bone formation was set to 27% and based on this study, we assumed the standard deviation on the relative change to be 8%. Two sided α and β were set to be 5% and 10% respectively.

All procedures were approved by our institutional Animal Ethics Committee.

### Bone graft preparation

Cancellous bone graft was obtained under sterile conditions from the sternum of three donor goats. Bone grafts were morselized into chips of approximately 1-2 mm, pooled and rinsed with saline with pulse lavage (Pulsavac^® ^Plus, Zimmer, Swindon, UK) for two minutes to remove blood and marrow.

Bone chips were impregnated with 5 ml alendronate (Fosamax, MSD) and/or 5 ml cefazolin solutions for 10 min, then rinsed 3 times in 5 ml saline at room temperature for 3 min. Rinsing was done to remove unbound and excessive alendronate and/or cefazolin. Alendronate solution was prepared by dissolving one 10 mg tablet in 5 ml of saline for 1 h and then passing it through a sterile Millipore filter with a pore size of 0.2 mm. To obtain the different concentrations, this alendronate solution was diluted.

The concentration of cefazolin was 200 μg/mL. This concentration was based on an earlier performed *in vitro *study, in which the amount of antibiotics on the allograft bone was determined after impregnation of the bone chips with cefazolin [35]. The amount of cefazolin present on the bone chips after rinsing 3 times with saline was determined and appeared to be well above the Minimal Inhibitory Concentration (MIC) for *S. Epidermidis *and about 1/3 of the amount of cefazolin present on the bone chips without rinsing. Additional data (unpublished) showed that impacting the bone graft chips after impregnation with cefazolin, did not have an effect on the amount of cefazolin present on the bone chips, compared to not impacting the bone chips. Tests were performed according to the protocol as described earlier [35]. According to Edin et al. [36], local levels of cefazolin of 200 μg/mL decrease cell replication, but local levels of 100 μg/mL do not affect the replication of osteoblasts. Freeze-dried DBM (size 80-800 μm) was prepared at DIZG (Deutsches Institut fur Zell- und Gewebeersatz, Berlin, Germany) from the cortical bone of two donor goat femora under sterile conditions. DBM was rehydrated and mixed with the cancellous bone graft chips in a 50/50 volume ratio.

Impaction was performed by gradually filling the chambers with the allograft preparations. A piston slightly smaller in diameter (1.9 mm) was used for impaction. The piston was guided by low friction bearings, strictly limiting it to vertical movement. During impaction, the chamber was clamped into a cylindrical holder. A constant force of 40 N was kept on the free end of the piston for two minutes. During this time, fluid could escape between the piston and the wall of the bone chamber and the ingrowth openings. The pressure applied was calculated to be 25 MPa. With this method of impaction, the mean volume fraction of graft bone in the bone chamber rises from about 35% in unimpacted grafts to about 65% in impacted ones [37]. After impaction, the screw cap was placed on the cylinder and the bone conduction chamber was stored at -80°C until use. The bone chambers were thawed at room temperature before implantation.

### Surgical procedure

Goats were anesthetized with medetomidine (5 μg/kg) and propofol (3-5 mg/kg), intubated, and maintained using isoflurane and oxygen in a semiclosed ventilation system. The goats were placed in supine position. The lower legs were shaved, washed, iodized and covered with sterile cloths. A longitudinal incision was made in the skin and fascia over the medial side of the proximal tibia. The cortical bone was explored and a hole was drilled through the medial cortex at approximately 4 cm from the joint cleft using a 3.2-mm drill. The hole was tapped and bone debris from drilling was removed. The bone conduction chamber was screwed in manually. The other three bone conduction chambers were placed 10 mm from each other. This was repeated for the other side. The skin was sutured in two layers.

After the operation, goats received injections of antibiotics (15 mg/kg) (ampicilline) and analgetics (1 mg/kg) (flunixin). All animals were allowed unrestricted movement in their cages and had free access to water and food after the operation. After 12 weeks, the goats were killed with an overdose of sodium pentobarbital (0.5 mL/kg).

### Histological and histomorphometric evaluation

Tibiae were removed and the bone conduction chambers with surrounding cortex were fixed in 4% buffered formalin. After 1 day the contents were removed from the bone chambers and fixed additionally for at least 5 days. After dehydration in ethanol and plastic embedding (polymethylmethacrylate), non-decalcified 7-mm thick serial sections were made along the longitudinal axis of the specimen. Sections were stained with hematoxylin and eosin and Goldner-Masson for routine histological analysis. Histomorphometric quantitative analysis was done blindly using digitalized pictures of the sections. Three central sections in each specimen were studied, each 250 μm apart. Manual point counting was performed using custom-made stereological software. The total number of points, points covering bone in general and points covering new living bone were counted. Graft bone was identified by its highly organized lamellas and oval cell lacunae without nucleic material, whereas newly formed bone was less organized and contained nucleic material. On average, 100 point were counted in each section, resulting in 300 points per specimen All sections were blinded and evaluated in random order.

### Statistics

Results were analysed non-parametrically, therefore Friedman's repeated measurements ANOVA on ranks with a Wilcoxon signed ranks test with a Bonferroni adjustment was used to differentiate between groups.

SPSS software version 18.0 was used. Statistical significance was set at p < 0.05.

## Results

All goats were able to stand normally on their hind legs 1 to 2 days after surgery. There were no signs of inflammation, skin ulceration or wound healing problems. Three goats died during the 12 week period, of reasons not related to surgery. These goats were not excluded from analysis, since they died a few days before sacrification. 8 bone chambers were loose and excluded, they originated from the control group (1), control with cefazolin group (2), 0.5 mg/mL alendronate group (1), 2 mg/mL alendronate group (1), 10 mg/mL alendronate group (2) and of the DBM group (1).

### Histology

**Figure 1:**
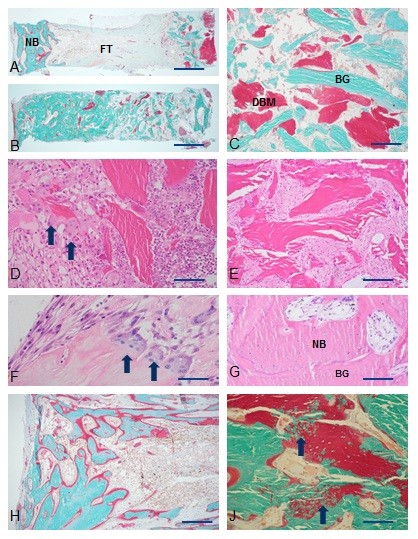
**Histological results**. A. Typical example of ALL + DBM group. Notice large area of fibrous tissue (FT) between graft remnants (right) and new bone (NB). B. Typical example of BIS + DBM group. Bone chamber is fully filled with a mixture of graft remnants, DBM and new bone. C. Non incorporated mixture of bone graft (BG) and demineralized bone (DBM) of ALL + DBM + BIS group. D. The incorporation front in a chamber with ALL. Arrows point at osteoclasts. E. Fibrous tissue surrounding bone grafts. ALL + AB + 2BIS group. F. Osteoclasts on DBM bone particle. ALL + DBM group. G. Enlargement of bone in ALL + AB + 2BIS group. Notice mixture of new bone (NB) with osteocytes and bone graft (BG). H. New bone formed in ALL + DBM group. J. Ossification (arrows) of BIS particles in bone chamber of ALL + DBM + BIS group. Bars are 1 mm (A, B), 500 μ (H), 250 μ (C, D, E, J) or 100 μM (F, G).

In general, bone chambers were surrounded by a layer of callus and covered with fibrous tissue. The graft bone in the control group was almost entirely resorbed (Figure 1A), while in the alendronate groups the bone chambers were full with graft bone as well as new bone (Figure 1B). A homogeneous distribution of DBM was present in non-resorbed areas within the bone chamber (Figure 1C). Two different incorporation patterns were found in the ALL, ALL + AB, ALL + DBM groups on the one hand (Figure 1A) and all the other groups where BIS was added on the other hand. (Figure 1B).

In all groups without BIS (ALL, ALL + AB, ALL + DBM) similar patterns of resorption and new bone formation were present. All bone graft (including DBM if present) was resorbed or a resorption front could be found at the interface with allograft remnants (Figure 1D). This front, if still present, consisted of numerous lymphocytic cells that had penetrated into the graft (Figure 1D). Numerous osteoclasts in a blood vessel rich area resorbed all allograft and DBM if present (Figure 1D). No obvious differences were found between the activity of osteoclasts on allograft bone or DBM (Figure 1D, F). Between the resorption front and the area with new bone in almost all chambers a thick fibrous interface was present containing fibroblasts and some macrophages (Figure 1A). New bone formation was mainly found at the direct vicinity of the ingrowth holes (Figure 1A, H). The newly formed bone contained no or scarce remnants of ALL or DBM. In most chambers a strong remodeling activity was still present as is visualized by the red non-calcified zones in the Goldner stained sections (Figure 1J).

In all groups with BIS the delineation of the resorption front was less clear. Lower numbers of osteoclasts were present on the bone graft particles (Figure 1E) compared to all groups without BIS. Histologically no clear differences could be found in the number of osteoclasts in the different BIS concentration groups. After the passage of the revascularization front through the bone graft, the non-resorbed ALL and/or DBM were surrounded by fibrous tissue (Figure 1E). Newly formed bone was present in a much larger area compared to all groups without BIS (Figure 1B). Bone was formed on the remnants of ALL or DBM (Figure 1G, J). No clear differences were found between the resorption of ALL and DBM. In the area with new bone most allograft bone was mineralized. In the Goldner stained sections some focal areas with non-mineralized DBM were present, but round green spots in the DBM particles suggest that they underwent a process of mineralization after incorporation into new bone (Figure 1J).

### Histomorphometry

#### Cefazolin

**Figure 2:**
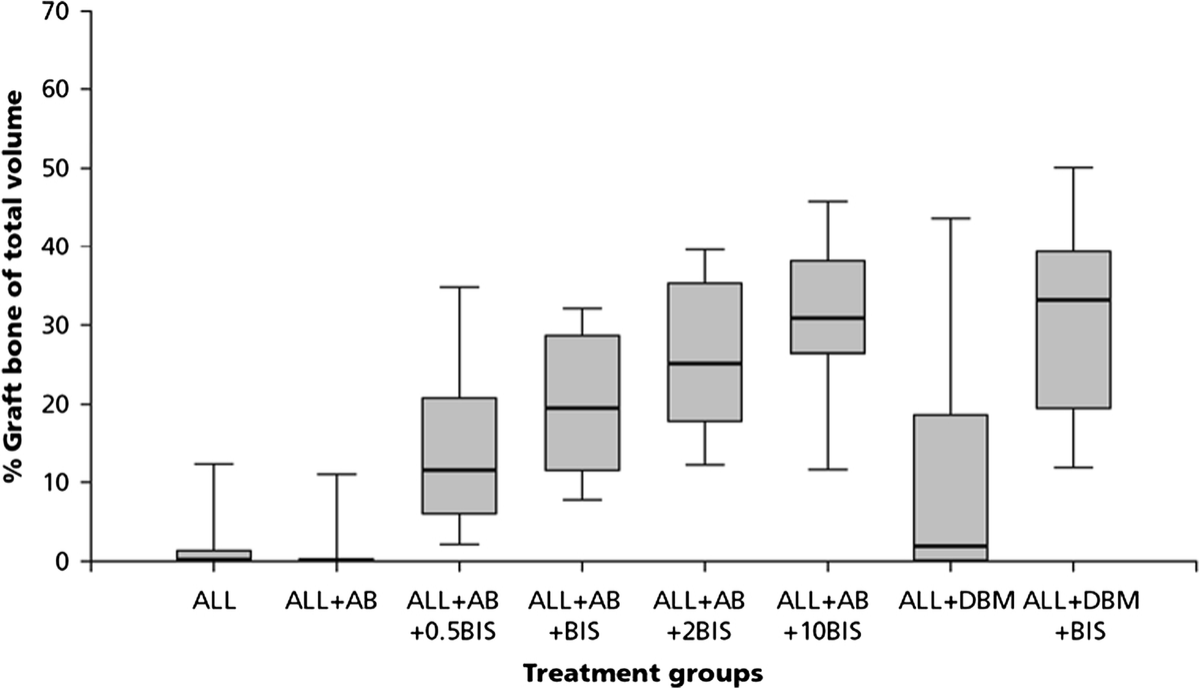
**Percentage graft bone of total volume for the 8 different groups**.

**Figure 3:**
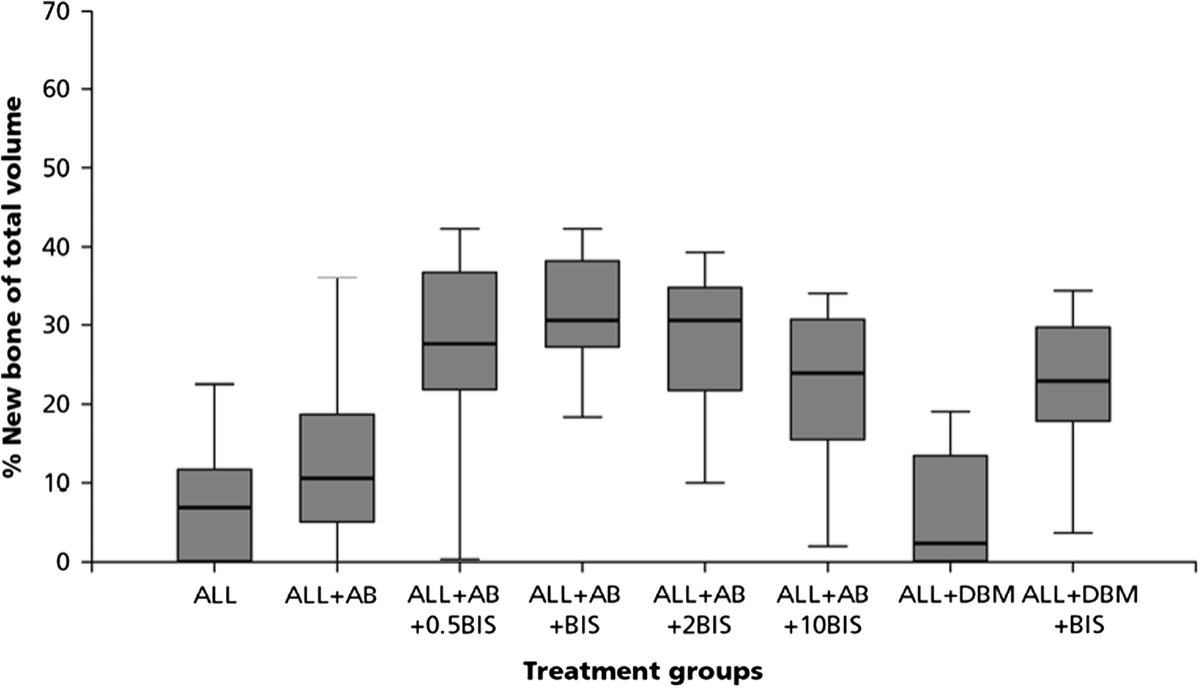
**Percentage new bone of total volume for the 8 different groups**.

No significant differences could be observed between the cefazolin group (ALL + AB) and the control group (ALL), for total bone volume, graft bone volume or new bone volume (Tables 2, and 3, Figure 2 and 3)

**Table 2:** Overview of statistical results (graft bone)

Group	ALL	ALL + AB	ALL + AB	ALL + AB	ALL + AB	ALL + AB	ALL+	ALL+
			
			+0.5BIS	+BIS	+2BIS	+10BIS	DBM	DBM + BIS
ALL	-	-	-	-	-	-	-	-

ALL + AB	0.674	-	-	-	-	-	-	-

ALL + AB+	< 0.001*	< 0.001*	-	-	-	-	-	-
								
0.5BIS								

ALL + AB+	< 0.001*	< 0.001*	< 0.001*	-	-	-	-	-
								
BIS								

ALL + AB+	< 0.001*	< 0.001*	< 0.001*	0.001*	-	-	-	-
								
2BIS								

ALL + AB+	< 0.001*	< 0.001*	< 0.001*	< 0.001*	0.003	-	-	-
								
10BIS								

ALL + DBM	0.003	0.003	< 0.001*	< 0.001*	< 0.001*	< 0.001*	-	-

ALL+	0.01	< 0.001*	0.000*	0.001*	0.003	0.36	< 0.001*	-
								
DBM + BIS								

*significant: p < 0.0018 (Bonferroni adjustment due to 28 different comparisons)

**Table 3:** Overview of statistical results (new bone)

Group	ALL	ALL + AB	ALL + AB	ALL + AB	ALL + AB	ALL + AB	ALL+	ALL+
			
			+0.5BIS	+BIS	+2BIS	+10BIS	DBM	DBM + BIS
ALL	-	-	-	-	-	-	-	-

ALL + AB	0.07	-	-	-	-	-	-	-

ALL + AB+	< 0.001*	0.06	-	-	-	-	-	-
								
0.5BIS								

ALL + AB+	< 0.001*	< 0.001*	< 0.001*	-	-	-	-	-
								
BIS								

ALL + AB+	< 0.001*	< 0.001*	0.007	0.219	-	-	-	-
								
2BIS								

ALL + AB+	< 0.001*	0.285	0.049	0.002	0.018	-	-	-
								
10BIS								

ALL + DBM	0.344	0.181	0.001*	< 0.001*	< 0.001*	< 0.001*	-	-
								
ALL+	< 0.001*	0.11	0.375	< 0.001*	0.043	0.331	< 0.001*	-
								
DBM + BIS								

*significant: p < 0.0018 (Bonferroni adjustment due to 28 different comparisons)

#### Alendronate

An alendronate dose-dependent difference in the amount of graft bone was found (Table 2, Figure 2). Graft resorption decreased with increasing alendronate concentration. All groups treated with alendronate differed significantly (p < 0.001) when compared to the volume fraction of allograft bone of the control group. These significant differences for volume fraction of allograft bone were also observed between the four alendronate-groups; with increasing amounts of alendronate, a decrease in graft resorption was found, which were all significantly different.

Bone formation in the four alendronate groups was significantly different when compared to the control group (p < 0.001) (Table 3, Figure 3).

When bone formation in the 1 mg/ml (ALL + AB + BIS) was compared to bone formation in the 10 mg/ml (ALL + AB + 10BIS) group, the volume fraction of new bone was significantly lower in the highest alendronate group (Tables 2, and 3).

#### Demineralized bone matrix

When the group with DMB (ALL + DBM) was compared to the control group, no differences could be observed (Tables 2, and 3).

## Discussion

The purpose of this study was to investigate the dose-response relationship of alendronate-impregnated allograft with respect to the amount of graft bone and the amount of new bone or total bone after 12 weeks in a goat bone chamber model. Also, the effects of adding DBM and the local application of antibiotics (cefazolin) were tested.

We showed a dose-response relation for local application of alendronate with respect to the amount of new bone as well as the amount of graft bone present in the bone chamber after 12 weeks. Decreased implant fixation has been observed with higher doses of bisphophonates (by blocking bone metabolism completely) [16]. Therefore, an optimum dose regarding bone resorption is essential, since it yields a positive balance between allograft resorption and the net amount of newly formed bone which results in improved fixation.

Our results show that with increasing alendronate concentration, graft resorption decreased and the amount of necrotic graft bone, left after 12 weeks, increases. This is in accordance with the study of Jakobsen et al. [20], in which another bisphosphonate, zoledronate, was used. They also studied different bisphosphonate concentrations, however their volume fractions of allograft bone were much higher compared to our study. Our volume fractions of allograft bone were 10.9% (± 8.8) for the lowest alendronate group to 32.8% (± 8.7) for the highest alendronate group, while in the study of Jakobsen, graft bone volume fractions started with mean values above 30%. These differences in results might also be explained by the rinsing method used (3 min vs 10 min) or the fact that Jakobsen had a mechanically loaded situation compared to our unloaded situation. Agholme et al. [17] also used alendronate locally (2 mg/mL) and found volume fractions of allograft bone compared to ours. In contrast to our study, they did not find any differences between the amount of new bone between a regular dose (with rinsing after impregnation) of bisphosphonate and an overdose amount (without rinsing the graft after impregnation). The volume fractions of new bone were comparable to the mean volume fractions of the 4 different doses in our study, although we did find differences between the doses. They stated that their results may be explained by the four-week study period in their experiments compared to the 6 weeks period in other studies (and thus the 12 week period in our study), that there was not enough time to fully resorb all allograft bone behind the bone ingrowth frontier.

Zoledronate is used in several studies for local application of bisphosphonate and is a more potent bisphosphonate compared to alendronate [38]. Differences in graft volume fractions between local application of alendronate and zoledronate might be explained by this. Alendronate was chosen in this study as bisphosphonate since it has been used as osteoporosis prophylaxis treatment for almost two decades [39].

The amount of new bone in our experiment showed an optimum at a dose of 1 mg/mL alendronate. The amount of new bone was significantly higher compared to control and also when compared to lower and higher alendronate concentrations.

A possible explanation for the difference in newly formed bone between control and BIS groups is that in the control groups the newly formed bone is rapidly remodeled by osteoclasts. This yields a net low amount of new bone, which is entirely the result of osteoclastic activity and might explain the differences observed. Jakobsen et al. stated that this effect could be explained by the preserving effect of the bisphosphonate on the allograft, thereby prolonging the osteoconductive effect [20].

An enhancing effect of application of bisphosphonate locally on the amount of new bone has also been observed in other studies [9,20,40,41] and are confirmed by some *in vitro *studies [19,42-44]. However, it has not been shown *in vivo*. The declination in the amount of newly formed bone at the highest alendronate-group,

compared to lower alendronate groups, might be explained by the toxicity of bisphosphonates on bone tissue. At a high dose, bisphosphonates have been shown to be toxic to osteoblasts [44,45]. Another explanation might be the lack of space in the bone conduction chamber. The necrotic bone is not resorbed and therefore, no room is left for the formation of new bone.

We chose cefazolin as antibiotic. This is the antibiotic of first choice for orthopaedic surgery in The Netherlands. According to Edin et al. [36] local levels of cefazolin 200 μg/mL decrease cell replication, but levels of 100 μg/mL do not affect the replication of osteoblasts. The amount of cefazolin used in this study is well above the MIC for *S. Epidermidis*, and did not affect bone remodelling since no differences between the control group and the allograft group impregnated with cefazolin could be observed between for % graft bone volume or % new bone volume. However, since our study was powered to show differences, not equality, this result should be interpreted cautiously. Taking into account the half-life of cefazolin, it will stay above MIC - and be effective- for at least 8 to 10 h which is enough for prophylaxis. Cefazolin is completely eluted from the bone chips after three days. No subinhibitory amount of the drug is left behind which can induce resistancies and therefore, cefazolin is an attractive choice for local prophylaxis [29].

No effect of the addition of DMB to allograft bone has been found in this study. In addition, histology shows similar resorption characteristics for DBM and allograft groups. The release of growth factors by osteoclastic resorption of allograft bone or DBM is probably quite similar. In addition, when combining DBM, alendronate and allograft bone, no additional bone was formed. Our results confirm several studies [46,47], although other studies did find an enhancing effect of DBM on bone formation [48,49]. Bae et al. [50] studied different DBM products and found a higher variability in concentration of bone morphogenic proteins among three different lots of the same DBM than among the different DBM products of different companies. Although our study pooled DBM of two goats (and therefore pooled two different lots), we did not find any effect of the addition of DBM to allograft bone.

Before implantation of the bone chamber we impacted the allograft bone to simulate the clinical situation as much as possible. However, caution should be taken when extrapolating the results. This bone chamber model in goats is un-loaded and therefore quite different from the loaded conditions as in a clinical situation. Also, no cement has been applied, as is done in a cemented revision hip arthroplasty with bone impaction grafting. In addition, a limitation of this study is the lack of biomechanical testing that would determine whether the groups treated with alendronate would be structurally stronger than the control group.

Basic principle of bone impaction grafting is that osteoclasts will resorb bone and osteoblasts will form new woven bone. Micromotions during normal gait cycles induce a rapid osteoclastic response. This too fast bone resorption might lead to intial instability of the implant, periprosthetic osteolysis and later implant migration [6,7,11,51]. Local application of bisphosphonates has a clear influence on the osteoclastic activity, but little is known of the effect on implant migration and the occurrence of micromotions surrounding the implant.

## Conclusions

In conclusion, a dose-response relationship for local application of alendronate with respect to graft resorption has been shown in this study. The area of new bone in our experiment was optimal at 1 mg/mL alendronate. At this concentration, the amount of new bone was significantly higher compared to control and also when compared to lower (0.5 mg/mL) and higher alendronate concentrations (2 mg/mL). Local application of cefazolin (200 μg/mL) had neither effect on bone formation nor on bone graft resorption. Therefore, cefazolin at this concentration might be used as a prophylaxis against infection. The addition of DBM did not enlarge the new bone area.

## Competing interests

The research study was funded by Bislife, The Netherlands

## Authors' contributions

NMCM carried out the experiments reported here, coordinated the study and drafted the manuscript. GH assisted with the experiments and participated in the design and coordination of the study. BWS en RMB participated in the design of the study. PB participated in the design of the study and assisted in drafting the manuscript. All authors read and approved the final manuscript.

## Pre-publication history

The pre-publication history for this paper can be accessed here:

http://www.biomedcentral.com/1471-2474/13/44/prepub
